# Value-based assessment of repeat surgery for recurrent glioma: A real-world quality-adjusted survival analysis

**DOI:** 10.1093/noajnl/vdag251

**Published:** 2026-09-18

**Authors:** Wanyu Qiao, Chen Luo, Tian Gan, Shuai Wu, Xian Xie, Jinsong Wu, Hugues Duffau, Jie Zhang, Ye Yao

**Affiliations:** Department of Biostatistics, School of Public Health, Fudan University, Shanghai; Shanghai Center for Health Technology Assessment, Shanghai; Glioma Surgery Division, Neurologic Surgery Department, Huashan Hospital, Shanghai Medical College, Fudan University, Shanghai; Shanghai Key Laboratory of Brain Function Restoration and Neural Regeneration, Huashan Hospital, Fudan University, Shanghai; Brain Function Laboratory, Neurosurgical Institute of Fudan University, Shanghai; Department of Biostatistics, School of Public Health, Fudan University, Shanghai; Glioma Surgery Division, Neurologic Surgery Department, Huashan Hospital, Shanghai Medical College, Fudan University, Shanghai; Shanghai Key Laboratory of Brain Function Restoration and Neural Regeneration, Huashan Hospital, Fudan University, Shanghai; Brain Function Laboratory, Neurosurgical Institute of Fudan University, Shanghai; Department of Biostatistics, School of Public Health, Fudan University, Shanghai; Glioma Surgery Division, Neurologic Surgery Department, Huashan Hospital, Shanghai Medical College, Fudan University, Shanghai; Shanghai Key Laboratory of Brain Function Restoration and Neural Regeneration, Huashan Hospital, Fudan University, Shanghai; Brain Function Laboratory, Neurosurgical Institute of Fudan University, Shanghai; Department of Neurosurgery, Gui de Chauliac Hospital, Montpellier University Medical Center, Montpellier; Institute of Functional Genomics, University of Montpellier, CNRS, INSERM, Montpellier; Glioma Surgery Division, Neurologic Surgery Department, Huashan Hospital, Shanghai Medical College, Fudan University, Shanghai; Shanghai Key Laboratory of Brain Function Restoration and Neural Regeneration, Huashan Hospital, Fudan University, Shanghai; Brain Function Laboratory, Neurosurgical Institute of Fudan University, Shanghai; Department of Biostatistics, School of Public Health, Fudan University, Shanghai; Key Laboratory of Public Health Safety of Ministry of Education, Fudan University, Shanghai

**Keywords:** glioma, logistic models, quality of life, reoperation, survival analysis

## Abstract

**Background:**

Reoperation for recurrent glioma is widely practiced, yet clinical decision-making remains challenging due to substantial trade-offs between survival gain and postoperative functional burden. High-quality real-world evidence integrating survival and quality-of-life outcomes to inform reoperation decisions remains limited.

**Methods:**

We conducted a retrospective real-world cohort study of 1304 patients with recurrent glioma treated between 2010 and 2018. Propensity score matching was applied to balance baseline characteristics. Survival outcomes, quality-adjusted life years (QALYs), functional status, and prognostic factors associated with reoperation benefit were evaluated.

**Results:**

In the 1:1 propensity-matched cohort (140 reoperation vs 140 non-reoperation), reoperation was associated with a mean gain of 9.6 months in quality-adjusted life years (QALYs) and a 521-day longer overall survival. Postoperative functional status showed short-term improvement. In the glioblastoma subgroup, reoperation was associated with a numerical gain of 3.5 QALY months, although the confidence interval crossed zero, alongside a 218-day gain in overall survival. Predictive models based on preoperative characteristics demonstrated good discrimination in identifying patients likely to derive survival benefit beyond 6 months.

**Conclusion:**

This study provides real-world evidence that carefully selected patients with recurrent glioma may derive quality-adjusted survival benefits from reoperation, thereby supporting individualized surgical decision-making. These findings have implications for value-based surgical decision-making in recurrent glioma, particularly in healthcare systems where balancing survival benefit against functional outcomes and resource use is increasingly important.

Key Points

- Reoperation at tumor recurrence in glioma patients shows survival benefit
- Reoperation at tumor recurrence in glioma improves postoperative quality of life.
- Preoperative clinical and pathological factors may help inform individualized reoperation decisions.

Importance of the Study

Tumor recurrence in patients undergoing initial glioma resection is an unavoidable challenge. The benefit of reoperation after recurrence remains elusive due to inherent selection bias and confounding factors. We retrospectively reviewed patients from an 8-year large-sample glioma cohort, using propensity score matching (PSM) methods to obtain a comparable control group, and analyzed the impact of reoperation from multiple perspectives, including survival and quality of life. Compared to patients who did not undergo reoperation, we found that reoperation, on average, improved OS by 521 days and QALYs by 9.6 months. Even among patients with glioblastoma, who generally have the poorest prognosis, median OS was 218 days longer, while QALYs showed a numerical increase of 3.5 months. Our findings may support further research aimed at better addressing the challenges faced by adult recurrent glioma patients in treatment decision-making, providing valuable insights to improve functional prognosis and optimize clinical practice.

Glioma, the most common primary CNS tumor, has a high incidence rate worldwide, with approximately 5.9 cases per 100 000 individuals and about 50% of patients diagnosed with WHO grade 4 tumors.^1^^,^^2^ Despite advancements in treatment and preoperative plans, the prognosis for glioma remains poor, and recurrence is inevitable.^3^^,^^4^ The 5-year survival rate for high-grade gliomas is less than 30%, and for glioblastomas, it is even less than 5%, resulting in a substantial loss of quality of life.^5^ Moreover, the risk of recurrence within 5 years for low-grade glioma patients who have undergone complete tumor resection exceeds 50%.^6^ These high rates of recurrence, disability, and mortality from glioma impose a substantial burden on patients’ families and society as a whole.

Reoperation for recurrent gliomas has been a long-standing treatment consideration.^7^^,^^8^ Despite this, the benefits of repeat surgery following recurrence remain elusive,^9^ with focused investigations on this matter only emerging recently.^10^^,^^11^ Several factors, such as tumor biological characteristics, selection bias, discrepancies in clinician criteria for patient selection, and inconsistent cohort results, constrain the utility of reoperation.^12-14^ Additionally, the impact of reoperation on neurological and physical functions, along with the risk of surgical complications, can significantly influence postoperative quality of life, an aspect that has been largely neglected in the past.^15^^,^^16^ Therefore, the potential benefits of reoperation must be judiciously evaluated against these possible drawbacks. Addressing this gap, preference-based health status measures such as EQ-5D-5L^17^^,^^18^ and the Karnofsky Performance Status (KPS)^19^ scores have been utilized to holistically assess the combined effects of reoperation on patients’ survival, overall condition, and quality of life, thereby facilitating more informed therapeutic decisions.

Prior attempts to evaluate the impact of reoperation on survival outcomes in glioma often suffered from an absence of comparable control groups and prognostically significant molecular markers, introducing confounding factors that muddled interpretation of results.^20^ Indeed, patient characteristics and treatment choices can mask actual treatment effects, especially in recurrent glioma management where preoperative KPS scores have a significant bearing on treatment decisions.^21^ PSM, a widely employed method in observational studies for causal inference, involves using specific statistical techniques to match the control and treatment groups, thereby facilitating bias minimization in treatment effect estimation and group differences adjustment.^22^ Recently, there has been growing interest in applying PSM to evaluate surgical treatment issues. Demonstrated efficacy in fields as diverse as neonatology and breast cancer research underscores PSM’s utility in mitigating confounding factors and creating more comparable groups.^23^^,^^24^ In glioma research, PSM could similarly offer a robust methodology to counter confounding factors, thus establishing more homogeneous treatment and control groups. Such as matching the above key factors that may affect the outcome to simulate a counterfactual scenario, the use of PSM is able to match more confounding factors than traditional matching, while achieving the effect of dimensionality reduction. Unlike traditional multivariate regression analyses that are constrained to a single outcome event, the PSM approach in our study allowed for multiple covariate control, creating uniformly comparable control groups. As a result, it enabled a more comprehensive comparison of outcome indicators under the same conditions. In particular, the value of PSM in observational studies needs to be emphasized more when the effect of reoperation as an intervention is difficult to assess in RCTs.

In this study, we conducted a propensity score-matched real-world cohort study of adults with recurrent glioma to evaluate the clinical value of reoperation. We integrated overall survival, quality-adjusted life years, and functional outcomes, examined molecularly defined subgroups, and developed exploratory models using routinely collected clinical and pathological variables known before reoperation. This comprehensive assessment was intended to characterize patients more likely to derive benefit from repeat surgery and to support individualized treatment decisions.

## Methods

### Study Design and Participants

This study included a total of 1304 patients who underwent postoperative recurrence after primary glioma resection at Huashan Hospital, Fudan University, between October 2010 and December 2018. The exclusion criteria for patients were (1) age <18 years, (2) having undergone 3 or more glioma resections, (3) having received biopsy only at initial surgery, and (4) missing information on follow-up, tumor locations, and WHO grades. Of the 1304 patients, 35 had undergone more than 2 tumor resections, 65 were under the age of 18, 40 had missing follow-up information, and 30 lacked WHO grades (as per WHO CNS tumor classification, version 2021) and tumor location information, and these patients were excluded from the study. The remaining 1134 participants formed the initial cohort, with 142 in the reoperation group and 992 in the non-reoperation group (Supplementary Figure S1). Decisions regarding reoperation were made by a multidisciplinary team comprising neurosurgeons, neuro-oncologists, anesthesiologists, and other relevant specialists. The assessment considered confirmation of tumor recurrence, surgical feasibility and neurological risk, KPS and neurological status, age and comorbidities, tumor location and molecular subtype, previous treatment, and potential subsequent therapeutic options. No single clinical or molecular factor was used as the sole criterion; the patient’s treatment goals, preferences, and willingness to accept surgical risks were also incorporated into the final decision. These considerations reflected our institutional clinical practice and were broadly consistent with published guidelines and reoperation studies.^13^^,^^25-27^ The study protocol was approved by the Ethics Review Committee of Huashan Hospital, Fudan University (No. KY2015-256). Written informed consent was obtained from all participants, and the study was conducted in accordance with STROCSS criteria.^28^

### Propensity Score Matching

To address selection bias and confounding factors, propensity score matching (PSM) was utilized to create counterfactuals and construct matched cohorts. A selection model using logistic regression was constructed to calculate propensity scores for each patient. Variables included age at diagnosis, sex, preoperative KPS, tumor location, and postoperative adjuvant treatment options. Furthermore, it was ensured that there was no statistical difference in the extent of resection between the 2 paired groups. Patients in the non-reoperation group were selected through a 1:1 nearest-neighbor matching with a caliper value of 0.2, resulting in a matched cohort of 280 patients (140 in each group).

Moreover, to balance between-group differences caused by physicians’ different treatment preferences and the trend toward sub-specialization in neuro-oncology, we first explored the effects of reoperation by subspecialists and non-subspecialists separately. Then, a thorough analysis of the HR of reoperations performed by the different surgical attending surgeons involved in the cohort was performed, and the HR of reoperations in the glioblastoma subgroup (according to the WHO CNS 5 classification criteria, only IDH wild-type) was also specifically analyzed. Finally, we subgrouped those patients in the non-reoperation and reoperation groups operated on by the same surgeon to balance the possible impact of different surgeons on outcomes.

### Patient Outcomes Measurement

The primary outcome measure was overall survival (OS), defined as the time from initial tumor resection to death from any cause. Secondary outcome measures included QALYs, KPS scores, and OS_2_ (overall survival after secondary surgery in reoperation patients). Progression-free survival (PFS) was defined as the time interval from first tumor resection to post-operative recurrence according to the Response Assessment in Neuro-Oncology criteria (RANO).^29^ The EQ-5D-5L asks about 5 dimensions of health (mobility; self-care; activity; pain; anxiety), with 5 levels of response (EQ-5D-5L: no problems, slight, moderate, severe, and extreme problems/unable to; best 11111, worst 55555).^17^ Utility values, reflecting the weighted preferences assigned to each health state within the general population of a specific country or region, are utilized in the calculation of quality-adjusted life years (QALYs).^30^ Many countries have established sets of utility index values for EQ-5D-5L tailored to their populations. We calculated QALYs using a set of utility values developed specifically for the Chinese population.^31^ The QALYs benefit of reoperation was computed as the mean difference between the QALYs of the reoperation and non-reoperation groups. KPS scores were used as a measure of general physical condition. Missing pre-reoperation KPS values were addressed in a sensitivity analysis using last observation carried forward (LOCF), with additional available-case analyses performed at each postoperative time point. Follow-up assessments were conducted regularly every 3 months initially and every 6 months after 1 year; see Supplementary Text for the detailed follow-up process.

### Predictive Models and Prognostic Analysis

In a matched dataset, the benefit of reoperation was assessed by comparing the differences in multiple outcome indicators including OS, QALYs, and KPS scores between the reoperation and non-reoperation groups. Moreover, we undertook a comprehensive investigation into the potential advantages of reoperation within distinct subgroups of IDH mutant oligodendroglioma, IDH mutant astrocytoma, and glioblastoma (according to the WHO CNS5 classification criteria, only IDH wild-type). A logistic predictive model was constructed to assess the survival benefit of secondary surgery beyond 6 months. The response variable was whether the OS benefit exceeded 6 months, a prespecified threshold selected for clinical interpretability. Sensitivity analyses were additionally performed using 3-, 9-, and 12-month thresholds. Furthermore,univariable and multivariable Cox proportional hazards analysis was conducted to identify positive and negative factors affecting the prognosis of patients undergoing reoperation for glioma.

### Statistical Analysis

Continuous variables were described by mean ± SD and median ± interquartile range (IQR), categorical variables were described by frequency (percentages) and comparisons between groups were made using Student’s *t*-tests, Chi-square tests, or Fisher’s exact test, as appropriate. The reoperation group was matched to the non-reoperation group on a 1:1 propensity score, with the matching type being nearest-neighbor matching and the caliper value set to 0.2. The balance check of the matched dataset was assessed using the XBalance function of the RItools package in R.^22^ The Kaplan–Meier survival curves of each group were plotted correspondingly, and the log-rank test was used to evaluate the survival differences between the groups. Performance of the logistic predictive model was estimated using the area under the curve (AUC) value of the receiver operating characteristic (ROC) curve. Nomograms were developed as a scoring prediction system for the survival benefit of reoperation beyond 6 months to facilitate clinical application. A stratified Cox proportional hazards analysis was used to find factors affecting the prognosis of reoperation patients. A threshold of *P* < .05 was considered to be a significant statistical difference.

QALYs were calculated using the following formula,^32^ and *n* denotes the number of time periods, *U_j_* denotes the patient’s health utility value at time period *j*, and *t_j_* denotes the length of the time interval.


$$\mathrm{QALYs}\;=\sum_{j=1}^{n}Uj.tj$$

The corresponding health utility values were plotted at each follow-up time point, and the scattered points were connected by a straight line, and the area under the curve was the patient’s QALYs. We assumed that the utility values remained constant over a period of 3-6 months, and the missing follow-up time points were filled using the most recent utility value. Confidence intervals for the differences in QALYs were calculated using bootstrap bias-corrected accelerated (BCa) intervals. The statistical analysis process was performed using R version 4.0.5 software.

## Results

### Patient Characteristics

The matched cohort formed after 1:1 propensity score matching included a total of 280 patients with recurrence after initial glioma resection, 140 each in the reoperation and non-reoperation groups. The matched cohort resulted in a well-balanced dataset with little variation in variables affecting prognosis between the 2 groups (*χ*^2^ = 5.69, *P* = .58). After matching, there were no statistical differences in age (mean [SD], 42.3 [12.4] vs 42.4 [11.8] years, *P* = .91), males (93/140 [66.4%] vs 80/140 [57.1%], *P* = .11), preoperative KPS scores (mean [SD], 86.9 [9.7] vs 85.5 [11.2], *P* = .25), initial extent of resection (complete or near-total/subtotal/partial: 44/43/17 vs 51/32/14, *P* = .34). Among patients with available preoperative tumor-volume data before the initial surgery, tumor volume was also comparable between the non-reoperation and reoperation groups (mean [SD], 55.79 [35.19] vs 49.77 [36.17], *P* = .24). There were also no significant differences in postoperative radiotherapy or chemotherapy (125/140 [89.3%] vs 122/140 [87.1%], *P* = .58), detailed treatment patterns (no radiotherapy or chemotherapy/radiotherapy alone/chemotherapy alone/combined radiotherapy and chemotherapy: 15/22/12/91 vs 18/14/23/85, *P* = .13), WHO Grades (Grades 1/2/3/4: 0/56/22/62 vs 0/55/27/58, *P* = .72) or most tumor locations between the non-reoperation and reoperation groups (Table 1, pre-matched patient characteristics are shown Supplementary Table S1).

**Table 1.** Demographic and clinical data for matched single and repeat resection patients

Demographic and clinical data	Single resection (*n* = 140)	Reoperation (*n* = 140)	*t*/*Z*/*χ* ^2^	*P*-value
Age at diagnosis (y)	42.3 (12.4)	42.4 (11.8)	0.12	.91
Sex male no. (%)	93 (66.4)	80 (57.1)	2.56	.11
Hospitalization days	19.7 (7.9)	18.3 (7.0)	1.34	.18
Preoperative KPS	86.9 (9.7)	85.5 (11.2)	1.14	.25
Chief complaints				
Headache no. (%)	59 (42.1)	64 (45.7)	0.36	.55
Dizziness no. (%)	16 (11.4)	24 (17.1)	1.87	.17
Nausea no. (%)	9 (6.4)	11 (7.9)	0.22	.64
Vomiting no. (%)	11 (7.9)	21 (15.0)	3.53	.06
Seizure no. (%)	10 (7.1)	19 (13.6)	3.12	.08
Intracranial space—occupying lesion or intracranial tumor^a^ no. (%)	7 (5.0)	5 (3.6)	0.35	.56
Impaired consciousness no. (%)	30 (21.4)	26 (18.6)	0.36	.55
Muscle weakness no. (%)	12 (8.6)	28 (20.0)	7.47	<.01**
Limb numbness no. (%)	7 (5.0)	16 (11.4)	3.84	.05
Speech disorder no. (%)	17 (12.1)	22 (15.7)	0.74	.39
Memory deterioration no. (%)	9 (6.4)	9 (6.4)	0.00	1.00
Visual impairment no. (%)	2 (1.4)	13 (9.3)	8.52	<.01**
Lethargy no. (%)	2 (1.4)	1 (0.7)	–^b^	1.00
History of head trauma no. (%)	3 (2.1)	1 (0.7)	–^b^	.62
Tumor characteristics and surgical features				
Frontal lobe no. (%)	68 (48.6)	79 (56.4)	1.73	.19
Parietal lobe no. (%)	15 (10.7)	23 (16.4)	1.95	.16
Occipital lobe no. (%)	9 (6.4)	11 (7.9)	0.22	.64
Temporal lobe no. (%)	57 (40.7)	51 (36.4)	0.54	.46
Insular lobe no. (%)	24 (17.1)	9 (6.4)	7.73	<.01**
Tumors of deep cerebral structures^a^ no. (%)	12 (8.6)	7 (5.0)	1.41	.23
Preoperative tumor volume at initial surgery (cm³)	55.79 (35.19)	49.77 (36.17)	1.18	.24
Extent of resection^a^	44/43/17	51/32/14	2.18	.34
Complete or near total resection no. (%)	44 (42.3)	51 (52.6)	–	–
Subtotal resection no. (%)	43 (41.3)	32 (33.0)	–	–
Partial resection no. (%)	17 (16.3)	14 (14.4)	–	–
Molecular and pathological features				
WHO Grade 1/2/3/4	0/56/22/62	0/55/27/58	0.65	.72
WHO grade 1 no. (%)	0 (0.0)	0 (0.0)	–	–
WHO grade 2 no. (%)	56 (40.0)	55 (39.3)	–	–
WHO grade 3 no. (%)	22 (15.7)	27 (19.3)	–	–
WHO grade 4 no. (%)	62 (44.3)	58 (41.4)	–	–
GFAP+^a^ no. (%)	138 (99.3)	137 (100.0)	-^b^	1.00
OLIG2+^a^ no. (%)	125 (91.2)	119 (88.8)	0.45	.50
TP53+^a^ no. (%)	110 (80.3)	98 (72.1)	2.55	.11
IDH-1/IDH-2+^a^ no. (%)	53 (40.5)	69 (49.3)	2.13	.14
MGMT+^a^ no. (%)	77 (57.9)	84 (60.0)	0.12	.72
Ki-67^a^> 20% no. (%)	23 (16.5)	29 (21.5)	1.08	.30
Follow-up data 1 month after initial surgery				
Postoperative KPS	86.1 (16.4)	88.1 (15.0)	1.05	.29
Chemoradiotherapy no. (%)	125 (89.3)	122 (87.1)	0.31	.58
Chemoradiotherapy	15/22/12/91	18/14/23/85	5.71	.13
No radiotherapy or chemotherapy, no. (%)	15 (10.7)	18 (12.9)	–	–
Radiotherapy alone, no. (%)	22 (15.7)	14 (10.0)	–	–
Chemotherapy alone, no. (%)	12 (8.6)	23 (16.4)	–	–
Combined radiotherapy and chemotherapy, no. (%)	91 (65.0)	85 (60.7)	–	–
Postoperative clinical neurological function examination^a^	39/77/15/7/2	50/66/16/2/1	–^b^	.25
Obstacle 0 no. (%)	39 (27.9)	50 (37.0)	–	–
Obstacle 1 no. (%)	77 (55.0)	66 (48.9)	–	–
Obstacle 2 no. (%)	15 (10.7)	16 (11.9)	–	–
Obstacle 3 no. (%)	7 (5.0)	2 (1.5)	–	–
Obstacle 4 no. (%)	2 (1.4)	1 (0.7)	–	–
ECOG grade^a^	31/83/18/7/1	36/76/18/5/0	–^b^	.82
Grade 0 no. (%)	31 (22.1)	36 (26.7)		–
Grade 1 no. (%)	83 (59.3)	76 (56.3)		–
Grade 2 no. (%)	18 (12.9)	18 (13.3)		–
Grade 3 no. (%)	7 (5.0)	5 (3.7)		–
Grade 4 no. (%)	1 (0.7)	0 (0.0)		–
	621	742		
Progression-free survival^c^ (PFS) (days)	541-763	671-896	−1.37	.17

a The majority of intracranial space—occupying lesion or intracranial tumor were discovered incidentally through accidental injury seeking medical attention or physical examination. Tumors of deep brain structures including the corpus callosum, Thalamus, Basal ganglia, Cerebellum, Ventricle, Saddle area, Brainstem, and other areas. Owing to missing high-quality MRI images, the extent of resection was obtained in 201/280 patients, classified according to the 2021 version of the RANO criteria; GFAP+, glial fibrillary acidic protein positive; OLIG2+, oligodendrocyte transcription factor 2 overexpression; TP53+, tumor suppressor gene p53 mutation; IDH-1/IDH-2+, isocitrate dehydrogenase 1 or isocitrate dehydrogenase 2 mutation; Ki-67, cell proliferation antigen Ki-67; MGMT+, O-6-methylguanine-DNA methyltransferase promoter methylation; EGFR+, epidermal growth factor receptor mutation. Postoperative clinical neurological function examination is an assessment of the patient’s neurological function by clinicians based on family and work autonomy and neurological dysfunction examination, and is divided into 5 grades from no to severe disorders according to severity. ECOG Grade refers to a scoring system that measures the extent to which cancer affects a patient’s daily living abilities (performance status) on a scale ranging from 0 (fully active) to 5 (dead). Glioblastoma, according to WHO CNS 2021 criteria, includes only IDH wild type.

b Fisher’s exact test was used when any expected cell count was <5; therefore, no test statistic is reported for Fisher’s exact tests.

c Progression-free survival (PFS) was defined as the time interval from first tumor resection to recurrence. The table shows the median PFS and its 95% confidence intervals for the 2 groups, and a nonparametric 2-sample procedure was implemented using the controlTest package in R to compare the median PFS.

** *P* < 1 × 10^−2^_._

Chief complaints recorded before the initial surgery were compared as baseline clinical characteristics between the 2 matched groups. Headache was the most common symptom, observed in 123 (43.9%) patients, a chief complaint of seizure was found in 29 (10.4%) patients, while Muscle Weakness (12/140 [8.6%] vs 28/140 [20.0%], *P* < .01) and Visual Impairment (2/140 [1.4%] vs 13/140 [9.3%], *P* < .01) appeared to be more frequently reported in the reoperation group. The WHO grades and all molecular markers such as IDH mutation and MGMT status did not differ between the 2 groups. The follow-up data 1 month after the initial surgery showed no statistical differences in functional and general status indicators such as postoperative KPS scores (mean [SD], 86.1 [16.4] vs 88.1 [15.0], *P* = .29), Postoperative clinical neurological function tests (Obstacles 0/1/2/3/4: 39/77/15/7/2 vs 50/66/16/2/1, *P* = .25), and Eastern Cooperative Oncology Group (ECOG) Grade (Grades 0/1/2/3/4: 31/83/18/7/1 vs 36/76/18/5/0, *P* = .82) between the 2 groups. Results showed no statistical difference in PFS between the 2 groups.

### Survival Analysis of the Matched Cohort

Table 2 shows the different survival benefits of reoperation for the matched cohort and for subgroups with different histological types. Figure 1 shows that in glioma patients, OS was much longer in the reoperation group than in the non-reoperation group, with median OS of 1482 and 961 days (*χ*^2^ = 22.1, *P* = 2.61 × 10^−6^) in the 2 groups, respectively. As shown in Figure 2, the benefits of reoperation are evident in different subgroups. The median OS was 693 and 911 days (*χ*^2^ = 7.7, *P* = 5.49 × 10^−3^) in the non-reoperation group compared to the reoperation group for the glioblastoma subgroup (IDH-wildtype). The median OS was 1464 and 1825 days (*χ*^2^ = 3.8, *P* = .05) for the IDH mutant astrocytoma subgroup. Median survival was 991 days in the IDH mutant oligodendroglioma non-reoperation group, and a valid estimate of median survival was not available for the reoperation group because they did not achieve more than 50% of outcome events during the study period. We also measured the OS_2_ in reoperation patients of the different subgroups, which was 460, 363, and 465 days for the total population, glioblastoma (IDH-wildtype), and IDH mutant astrocytoma subgroups, respectively.

**Figure 1.**
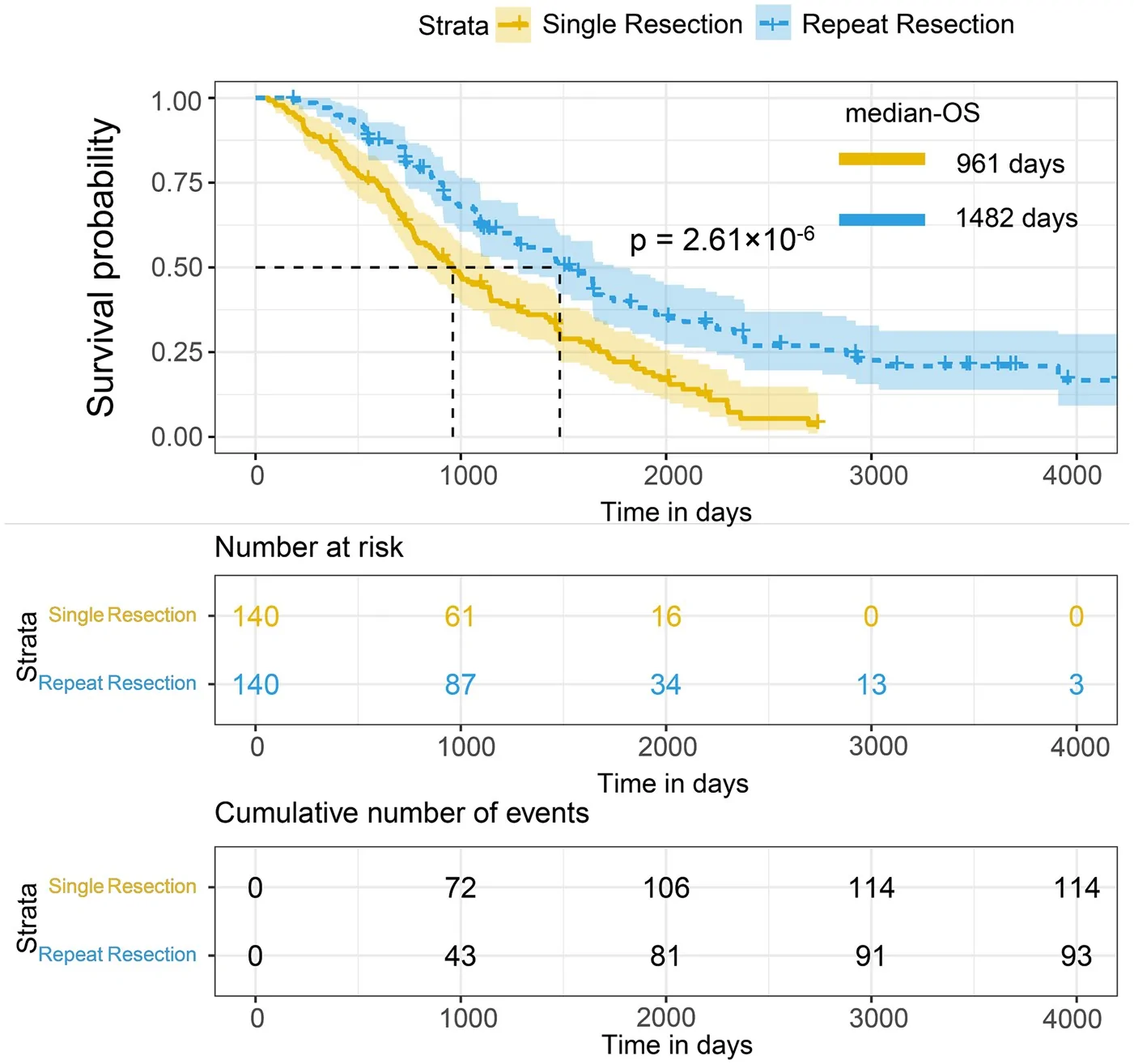
Kaplan–Meier survival curve between the reoperation and non-reoperation groups in the matched cohort. The matched cohort was formed by propensity score matching (PSM), and the selection model included age, sex, recurrence or not, preoperative KPS score, tumor location, chemotherapy or not, and WHO grades.

**Figure 2.**
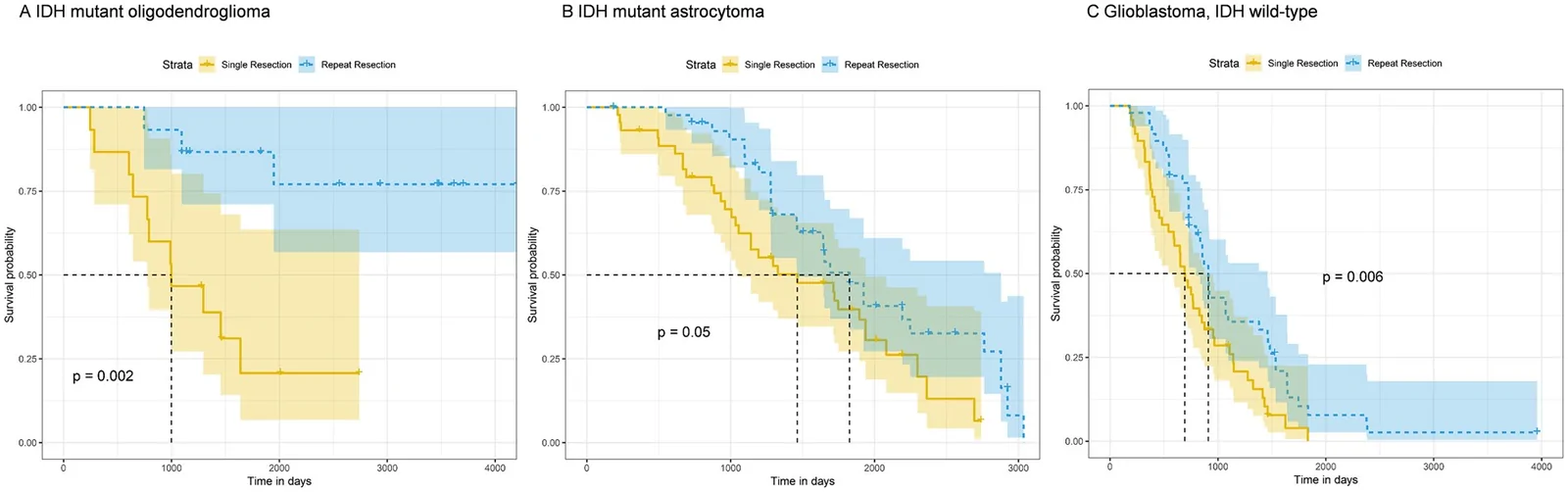
Kaplan–Meier survival curve between the reoperation and non-reoperation groups in different subsets. Kaplan–Meier survival curves for different subgroups of overall survival: part (A) is the IDH mutant oligodendroglioma subgroup, part (B) is the IDH mutant astrocytoma subgroup, part C is the glioblastoma subgroup.

**Table 2.** Survival outcomes within the matched population and subgroup population

Matched population	Single resection (*n* = 140)	Reoperation (*n* = 140)	**χ^2^** ^a^	*P*-value
OS (days)				
Median	961	1482	22.1	2.61 × 10^−6^ ***
95% CI	791-1148	1278-1745		
OS_2_ ^b^ (days)				
Median	–	460	–	–
95% CI	–	392-542	–	–

Glioblastoma^b^ subgroup	Single resection (*n* = 48)	Reoperation (*n* = 48)	*χ* ^2^ ^a^	*P*-value
OS (days)				
Median	693	911	7.7	5.49 × 10^−3^ **
95% CI	590-875	798-1460		
OS_2_ ^b^ (days)				
Median	–	363	–	–
95% CI	–	293-475	–	–

IDH mutant astrocytoma subgroup	Single resection (*n* = 44)	Reoperation (*n* = 44)	*χ* ^2^ ^a^	*P*-value
OS (days)				
Median	1464	1825	3.8	.05*
95% CI	1056-1937	1461-2879		
OS_2_ ^b^ (days)				
Median	–	465	–	–
95% CI	–	374-736	–	–

IDH mutant oligodendroglioma	Single resection (*n* = 15)	Reoperation (*n* = 15)	*χ* ^2^ ^a^	*P* value
OS (days)				
Median	991	NA^b^	9.9	1.69 × 10^−3^ **
95% CI	775-NA	NA		
OS_2_ ^b^ (days)				
Median	–	NA	–	–
95% CI	–	766-NA	–	–

a *χ* ^2^ is a statistic calculated by log-rank test.

b OS_2_ is defined as the time from the second surgery to death from any cause for reoperation patients. Glioblastoma according to 2021 WHO CNS5 criteria, including only IDH wild-type.

NA is due to the fact that more than 50% of patients in the IDH oligodendroglioma subgroup have not yet reached the study endpoints, as median survival could not be calculated.

* *P* < .05,

** *P* < 1 × 10^−2^,

*** *P* < 1 × 10^−3^.

The results from the neuro-oncology subspecialization showed that both subspecialists and non-subspecialists performing reoperations had a significant OS improvement in patients (Supplementary Figure S2a and S2b). The forest plots demonstrate the HR of reoperations by different surgeons (Supplementary Figure S3a), and the protective effect of reoperations on patient OS in the glioblastoma subgroup (Supplementary Figure S3b). The results of the subgroup of surgeries performed by the same surgeon showed a median OS of 961 and 1642 days for the non-reoperation group versus the reoperation group, respectively (Supplementary Figure S4).

### KPS Scores and QALYs of the Matched Cohort and Different Subgroups

KPS scores (mean [SD], 86.1 [16.4] vs 88.1 [15.0], *P* = .29) after the first surgery were not significantly different between the non-reoperation and reoperation groups (Table 3). The results showed that the preoperative KPS score (mean [SD], 78.5 [9.1] vs 85.5 [11.2], *P* = 4.57 × 10^−7^) was significantly lower in the second surgery than in the first. However, a trend toward higher KPS scores (mean [SD], 86.2 [17.1] vs 78.5 [9.1], *P* = 7.88 × 10^−3^) at 1 month postoperatively compared to preoperatively was found at follow-up. We also found that this trend became less pronounced over time, with a loss of statistical difference in KPS scores at 6 months postoperatively compared to preoperatively (mean [SD], 82.9 [18.9] vs 78.5 [9.1], *P* = .16). A sensitivity analysis using LOCF imputation yielded findings consistent with the primary analysis, showing significant short-term improvement in KPS at 1 month but no significant difference at 6 months after reoperation (Supplementary Table S3). In the matched cohort, the reoperation group had a mean QALY gain of 9.60 months compared with the non-reoperation group (95% CI, 3.35, 15.66). In the poorer prognostic glioblastoma subgroup (IDH-wildtype), the estimated QALY gain was 3.47 months (95% CI, −2.54, 8.81), although the confidence interval crossed zero.

**Table 3.** KPS scores and gained QALYs in matched cohort and different subgroups

Performance status comparison (KPS)	Single resection (*n* = 140)	Reoperation (*n* = 140)	*t*	*P*-value
KPS before primary surgery				
Mean (SD)	86.9 (9.7)	85.5 (11.2)	1.14	.25
KPS after primary surgery				
Mean (SD)	86.1 (16.4)	88.1 (15.0)	1.05	.29
KPS before reoperation				
Mean (SD)	–	78.5 (9.1)^a^	5.43	4.57 × 10−7^***^,^b^
KPS 1 month after reoperation				
Mean (SD)	–	86.2 (17.1)^c^	2.72	7.88 × 10−3^**^,^d^
KPS 6 month after reoperation				
Mean (SD)	–	82.9 (18.9)^c^	1.41	.16^e^

QALY gained by reoperation	Mean difference (months)	Improvement ratio^f^ (%)	95% CI^g^
Matched population	9.60	34.98%	(3.35, 15.66)
Glioblastoma subgroup	3.47	21.12%	(−2.54, 8.81)
IDH mutant astrocytoma subgroup	4.92	11.59%	(−4.51, 14.60)
IDH mutant oligodendroglioma subgroup	42.13	60.54%	(23.78, 64.48)

a Calculated from 50 of 140 patients with documented KPS before reoperation.

b From KPS scores before reoperation compared to KPS at initial surgery in 50 patients.

c Calculated from 50 of 140 patients with documented KPS 1 month after reoperation.

d From KPS scores before reoperation compared to short-term after reoperation in 50 patients.

e From KPS scores before reoperation compared to long-term after reoperation in 50 patients.

f The quality of life improvement ratio was calculated as the difference between the mean QALYs of the treatment and control groups divided by the mean QALYs of the control group.

g The confidence interval is calculated by the bootstrap method, and the bootstrap bias-corrected accelerated (BCa) is chosen.

** *P* < 1 × 10^−2^,

*** *P* < 1 × 10^−3^.

### Prognostic Factors for Patients on Re-Operation

Supplementary Table S4 shows the results of univariable and multivariable Cox regression analyses in the reoperation population. Variables were included in the multivariable model based on univariable results and clinical relevance. Male (HR, 1.93; 95% CI, 1.20, 3.10; *P* < .01) was risk factor affecting the prognosis of patients undergoing secondary surgery. In addition to this, the time interval between the 2 surgeries was an important influencing factor, with an HR of 0.55 (95% CI, 0.30, 0.99; *P* = .05) for patients with a time interval between the 2 surgeries of 365-730 days, compared with patients with a time interval between the 2 surgeries of less than 1 year. The association was stronger for a time interval between 2 surgeries of > 730 days, with an HR of 0.10 (95% CI, 0.05, 0.18; *P* < .001).

To further quantify the survival benefit beyond 6 months in patients undergoing reoperation, a logistic prediction model was developed using available data from the first operation. Variables with *P* < .05 in the univariate analysis were included in the multifactorial model, and the ROC curves showed predictive accuracy with an AUC value up to 0.91 (Figure 3A) and 0.88 (Figure 3B) in the matched cohort and glioblastoma subgroup. In the matched cohort, the interval between operations (OR, 1.004; 95% CI, 1.002, 1.006; *P* < .001) was the most important predictor (Supplementary Tables S5 and S6). Threshold sensitivity analyses showed that the interval between operations remained positively and significantly associated with survival benefit across the 3-, 6-, 9-, and 12-month definitions in both the matched cohort and the glioblastoma subgroup. Supplementary Figures S6 and S7 show the nomogram based on the high-precision prediction model for the matched cohort and glioblastoma subgroup in order to help physicians and patients make individualized treatment decisions.

**Figure 3.**
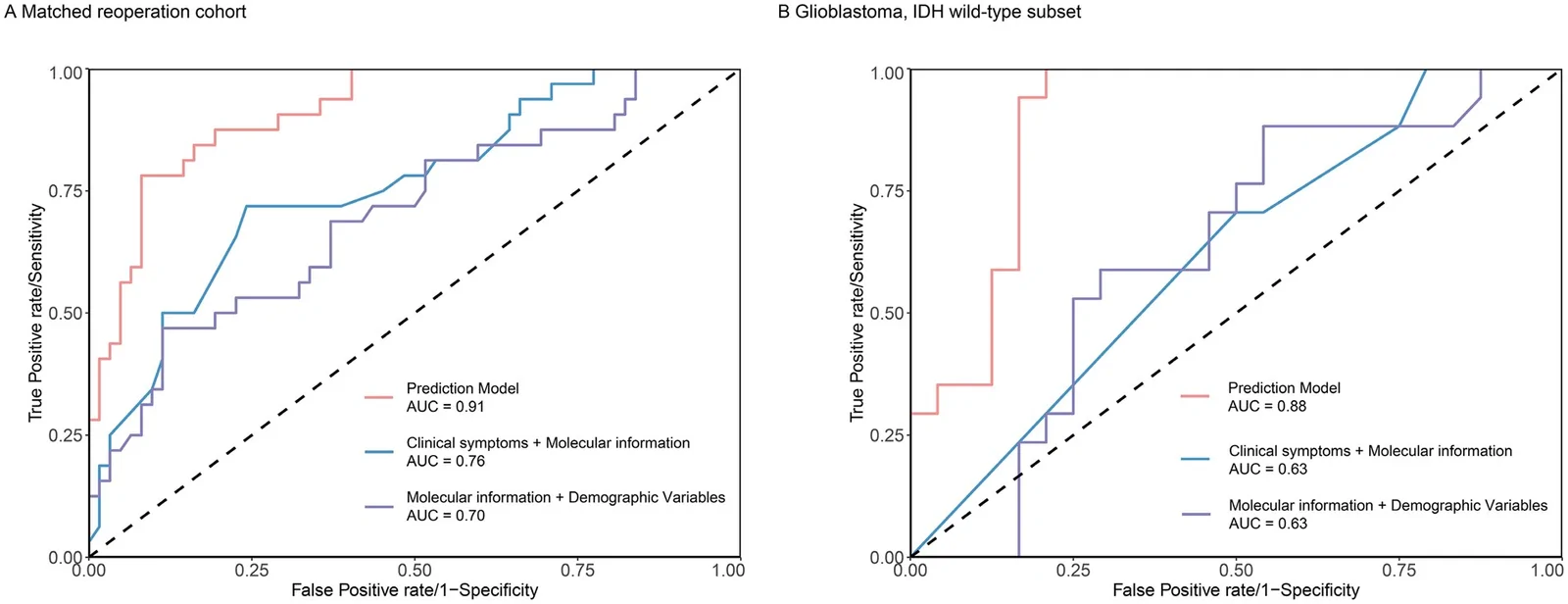
ROC curves for reoperation patients with matched cohort and glioblastoma subset. Receiver operating characteristic (ROC) curves were generated for matched (A) and glioblastoma (B) patients based on multiple logistic regression. Factors of initial surgery, such as age, sex, molecular information, etc., were included in the model to predict greater than 6-month survival benefit for reoperation patients versus non-reoperated. In panel (A), the full prediction model achieved an AUC of 0.91, while the clinical-symptom plus molecular model and the demographic plus molecular model achieved AUCs of 0.76 and 0.70, respectively. In panel B, the corresponding AUCs were 0.88, 0.63, and 0.63.

## Discussion

Patients who undergo initial tumor resection of gliomas face the challenging dilemma of inevitable recurrence.^33^ Various treatment modalities, including repeat surgery, re-radiation, and immunotherapy, have been employed to manage recurrent glioma cases.^34^^,^^35^ In recent years, there has been a growing emphasis on further surgical resection.^3^^,^^25^^,^^36^ However, the full extent of the benefits in terms of postoperative survival and quality of life for patients undergoing repeat resections remains unclear.^35^ Although randomized controlled trials remain the gold standard in clinical research, their feasibility and cost-effectiveness are often limited, particularly in assessing surgical interventions.^37^ PSM, a widely employed method in observational studies for causal inference, involves using specific statistical techniques to match the control and treatment groups, thereby ensuring comparability between the 2 groups in terms of potential confounding factors.^38^ Multiple factors, such as age and WHO Grades, can influence treatment choice for patients, and failure to adjust for these can distort the estimation of the true treatment effect.^39^ Unlike traditional multivariate regression analyses that are constrained to a single outcome event, the PSM approach in our study allowed for multiple covariates control, creating uniformly comparable control groups. As a result, it enabled a more comprehensive comparison of outcome indicators under the same conditions.

Our findings demonstrate a substantial improvement in OS among matched glioma patients who underwent repeat resection compared to those who did not. Considering the importance of molecular subtypes for tumor classification and prognosis, as emphasized in the fifth edition of the World Health Organization Classification of Tumors of the Central Nervous System (WHO CNS 5),^40^ we conducted a subgroup analysis based on different molecular and histopathological subtypes. Remarkably, even in patients with the poorest prognosis, such as those with glioblastoma, repeat resection extended OS by 218 days compared to non-reoperation patients. This finding is crucial given that all treatments for glioblastoma patients are palliative and carry the risk of side effects and mortality.^41^ Therefore, our results provide compelling evidence supporting selective reoperation in glioblastoma patients while considering the impact on quality of survival. An apparent survival advantage was also observed in the IDH-mutant oligodendroglioma and IDH-mutant astrocytoma subgroups. However, the oligodendroglioma subgroup included only 30 matched patients, and median OS was not reached in the reoperation arm. Given the limited number of events and the characteristically prolonged survival of this subtype, the survival and QALY estimates should be considered exploratory and interpreted cautiously. We developed a prediction model based on preoperative information, incorporating histopathological factors, which demonstrated the interval between operations as the most significant predictor, consistent with previous studies.^13^^,^^42^^,^^43^ This association is biologically plausible because a shorter interval to recurrence likely reflects more aggressive tumor behavior and, consequently, poorer prognosis. Therefore, the interval between surgeries should be interpreted primarily as a clinical marker of tumor aggressiveness rather than as an independently protective factor. This prediction model and the scoring system derived from it, with AUCs of 0.91 and 0.88 for the matched cohort and the glioblastoma subgroup, respectively, serve as a noninvasive tool for physicians and patients’ families to make treatment decisions from a preoperative perspective. However, it is important to exercise caution as most of our cases involve cerebral lesions, with only a few tumors located in deep structures such as the thalamus and brainstem. In such cases, the benefit of reoperation is not clear due to the potentially high risks associated with the procedure.

Our study, as illustrated in Supplementary Figures S2 and S3, reveals a noteworthy observation: both subspecialists in glioma surgery and general neurosurgeons achieved a significant improvement in overall survival (OS) when performing reoperations on patients. This is particularly relevant given the increasing trend of subspecialization within the surgical community over the past decade.^44^ Recent years have seen subspecialty trends in the field of surgery becoming the normal in healthcare as whole, and a similar trend has been seen in neurosurgery.^45^^,^^46^ In addition, many neurosurgeons gravitate toward a particular subspecialty out of choice. The reasons behind this are varied and include the natural tendencies of the surgical surgeon, specialty training, technical competence, and research interests, and academic departments may also encourage sub-specialization by faculty. There have been studies showing the impact of subspecialty trends on prognosis, and our hospital delineated subspecialty backgrounds for neurosurgeons. Subspecialized glioma surgeons typically receive more extensive training in glioma surgery and perform a higher volume of glioma resections compared to non-subspecialized surgeons. This likely indicates a higher level of specialization and greater proficiency in surgical techniques. In comparing the results of reoperations performed by glioma subspecialists and general neurosurgeons, we observed an intriguing pattern. While glioma subspecialists did exhibit better outcomes overall, this improvement was not statistically significant. It is crucial to interpret these preliminary findings with caution. Glioma subspecialists may tend to manage patients with more complex or severe conditions, thereby introducing a selection bias that could skew the comparison with general neurosurgeons.^45^ This potential bias serves as a limitation to our study and suggests the need for further, more rigorous investigation.

Another crucial aspect to consider when deciding on reoperation is the impact on the patient’s quality of life,^47^ and our positive results in terms of QALYs strongly support the case for reoperation. On average, reoperation improved QALYs by 9.6 months. When expressed in equivalent time units, the 9.6-month QALY gain represented approximately 55% of the 521-day (17.4-month) overall survival gain, suggesting that a substantial proportion of the survival benefit associated with reoperation was accompanied by preserved or improved quality-adjusted survival. In the glioblastoma subgroup, reoperation showed a numerical improvement of 3.47 QALY months, although the uncertainty of this estimate warrants cautious interpretation. For carefully selected patients, reoperation significantly enhances their quality of life, consistent with findings from previous studies.^8^^,^^36^^,^^48^ Further, in the study by Duffau et al,^49^ functional outcome remained better even in patients with third resection, ensuring quality of life. With advancing technology like awake craniotomy and intraoperative MRI (iMRI) detection, there is a greater desire for treatment to bring about not just prolonged survival but quality of life.^50^ In the latest study by Duffau et al in 2024,^51^ it was found that awake functional-guided surgery is an effective strategy for IDH-mutant low-grade gliomas, achieving long-term survival (>20 years) and functional independence (14.7 years). Merely using OS or PFS as reoperation treatment benchmarks is inadequate; considering post-surgery quality of life and overall functional status is equally crucial for patients.^52^ Postoperative KPS scores among reoperation patients further supported our findings. Even patients exhibited significantly poorer KPS scores before the second surgery compared to the first; a trend toward improved KPS scores in the short term after reoperation was observed. Multiple objective measures of functional outcome, reflecting changes in patient function or sensation, support the positive impact of reoperation on the functional outcomes of recurrent glioma patients.

### Limitation

Several limitations should be acknowledged. First, the retrospective design resulted in missing data for some molecular markers and pre-reoperation KPS scores, and residual bias related to incomplete functional-status data cannot be excluded despite sensitivity analyses. KPS is also a relatively coarse measure that may not fully capture subtle neurological deficits. Future prospective studies should incorporate neuro-oncology-specific measures, such as the NANO scale, to provide a more comprehensive assessment of neurological function. Second, selection bias remains possible because patients selected for reoperation may not represent the full spectrum of recurrent glioma cases, particularly in this single-center cohort. The relatively high KPS scores in both matched groups likely reflect the clinical selection of patients with relatively preserved functional status for reoperation, followed by matching of non-reoperated patients with similarly favorable status. Therefore, the findings may be less generalizable to patients with substantial functional impairment. Although propensity score matching balanced measured confounders, residual confounding from incompletely measured or unmeasured factors may remain. Tumor volume before the initial surgery was available only for a subset of patients, while recurrent tumor volume was not routinely recorded; detailed information on adjuvant chemoradiotherapy, including timing, regimen, dose intensity, duration, and sequencing, was also not consistently available. Reoperation decisions may additionally be influenced by patient preferences, family considerations, and financial circumstances. Given the ethical and practical challenges of randomized trials, larger prospective multicenter studies with more complete imaging, treatment, and neurological-function data are warranted.

## Conclusion

Rather than demonstrating that reoperation should be universally pursued, our findings highlight the importance of quantifying clinical tradeoffs when considering repeat surgery for recurrent glioma. By integrating overall survival, functional outcomes, and quality-adjusted life years, this study provides decision-level evidence that clarifies which patients are more likely to derive meaningful net clinical benefit from reoperation in routine practice. Importantly, our analyses were intentionally grounded in clinically accessible variables that are routinely available at the time of surgical decision-making. Together, these findings support a more individualized and transparent approach to reoperation decisions in recurrent glioma, emphasizing value-based assessment over binary treatment comparisons, and may also be informative for other high-risk oncologic interventions in which potential survival gains must be balanced against functional cost and quality of life within real-world healthcare systems.

## Supplementary Material

vdag251_Supplementary_Data

## Data Availability

Data and materials are available on reasonable request.
