# Supplementary material for: Value-based assessment of repeat surgery for recurrent glioma: A real-world quality-adjusted survival analysis
Source: Neurooncol Adv. 2026 Sep 18;8(1):vdag251. doi: 10.1093/noajnl/vdag251 (PMC13649579; doi:10.1093/noajnl/vdag251)
Supplement: Supplementary file 1 [file vdag251_supplementary_data.docx]

**Supplementary Material**

**Value-based assessment of repeat surgery for recurrent glioma: a real-world quality-adjusted survival analysis**

**Table S1.** Demographic and Clinical Data of baseline statistics for unmatched single and repeat resection.

**Table S2** Percentage of Missing data on variables with single and repeat resection patients.

**Table S3** KPS scores before and after reoperation: LOCF sensitivity analysis and available-case analyses.

**Table S4** Univariable and Multivariable Cox Analysis of the Reoperation Population (n=133).

**Table S5** Multivariable Logistic Regression Models for Predicting Survival Benefit After Reoperation at Different Thresholds.

**Table S6** Multivariable Logistic Regression Models for Predicting Survival Benefit After Reoperation at Different Thresholds in glioblastoma subset.

**Figure S1** Flow chart of the study.

**Figure S2** Kaplan-Meier survival curve between the reoperation and non-reoperation groups in the subspecialty and non-subspecialty subgroups.

**Figure S3** Forest plots of hazard ratios and 95% CIs for reoperation survival rate by different surgeons involved in the cohort.

**Figure S4** Kaplan-Meier survival curve between the reoperation and non-reoperation groups in the same primary surgeon subgroup.

**Figure S5.** Kaplan-Meier survival curve for WHO grades in multivariate COX analysis.

**Figure S6** A novel scoring system to predict survival benefit beyond 6 months for glioma patients.

**Figure S7** A novel scoring system to predict survival benefit beyond 6 months for glioblastoma patients.

**Supplementary Text**

**1.** **Standard Follow-Up Procedure**

Our standard follow-up protocol was as follows: patients were included in the follow-up bank after glioma resection, and the first follow-up was performed at one month postoperatively, with the main follow-up including postoperative KPS score examination, postoperative clinical neurological function examination, and ECOG grade, as well as inquiring about the occurrence of any serious adverse events, and monitoring of outcomes such as recurrence and death, and so on. Moreover, we will also measure scales related to quality of life and brain tumor symptoms, such as the MDASI-BT questionnaire, according to the patient's own physical and mental condition. After experiencing the first follow-up one month postoperatively, patients will be followed up every three months for the first year and every six months after one year, as described above, until the patient's death.

**2. Methods for detecting Isocitrate dehydrogenase 1/2 (IDH1/2) status**

For IDH1/2 detection, we employed direct sequencing targeting: 1) Codon 132 of IDH1 and codon 172 of IDH2; 2) Using 10μm FFPE tumor sections with >70% tumor content; 3) DNA extraction involved proteinase K treatment in Tris-HCL buffer; 4) PCR amplification and sequencing were performed using BigDye Terminator technology; 5)All mutations were confirmed by duplicate sequencing.

**3. Methods for detecting O-methylguanine-DNA methyltransferase (MGMT) status**

For MGMT analysis: 1) Methylation-specific PCR was used to assess promoter methylation; 2) Analysis utilized genomic DNA from FFPE tissues; 3) Bisulfite modification was performed using the Methylamp™ kit; 4)Positive methylation was defined by signal detection in 12% polyacrylamide gel.

| **Table S1 Demographic and Clinical Data of baseline statistics for unmatched single and repeat resection** | | | | |
| --- | --- | --- | --- | --- |
| **Demographic and Clinical Data** | **Single resection**  **(n = 992)** | **Reoperation**  **(n = 142)** | **t/Z/χ2** | **p value** |
| Age at Diagnosis (Years) | 51.1 (13.1) | 42.1 (11.9) | 7.71 | 2.76×10^- 14***^ |
| Sex male No. (%) | 638 (64.3) | 81 (57.0) | 2.53 | 0.11 |
| Hospitalization Days | 19.1 (9.0) | 18.2 (7.1) | 0.91 | 0.36 |
| Preoperative KPS | 84.4 (11.2) | 85.5 (11.0) | 1.06 | 0.29 |
| **Chief complaints** | | | | |
| Headache No. (%) | 451 (45.5) | 65 (45.8) | 0.00 | 1.00 |
| Dizziness No. (%) | 118 (11.9) | 24 (16.9) | 2.40 | 0.12 |
| Nausea No. (%) | 44 (4.4) | 11 (7.7) | 2.28 | 0.13 |
| Vomiting No. (%) | 72 (7.3) | 21 (14.8) | 8.38 | 3.79×10^- 3**^ |
| Seizure No. (%) | 33 (3.3) | 19 (13.4) | 26.45 | 2.71×10^- 7***^ |
| Intracranial Space-Occupying Lesion or Intracranial Tumor^a^ No. (%) | 29 (2.9) | 6 (4.2) | 0.34 | 0.56 |
| Impaired Consciousness No. (%) | 130 (13.1) | 26 (18.3) | 2.41 | 0.12 |
| Muscle Weakness No. (%) | 215 (21.7) | 28 (19.7) | 0.18 | 0.67 |
| Limb Numbness No. (%) | 62 (6.2) | 16 (11.3) | 4.13 | 0.04^*^ |
| Speech Disorder No. (%) | 139 (14.0) | 22 (15.5) | 0.12 | 0.73 |
| Memory Deterioration No. (%) | 101 (10.2) | 9 (6.3) | 1.68 | 0.20 |
| Visual Impairment No. (%) | 46 (4.6) | 14 (9.9) | 5.76 | 0.02 |
| Lethargy No. (%) | 8 (0.8) | 1 (0.7) | 0.00 | 1.00 |
| History of Head Trauma No. (%) | 4 (0.4) | 1 (0.7) | 0.23 | 0.42 |
| **Tumor Location** | | | | |
| Frontal lobe No. (%) | 497 (50.1) | 79 (55.6) | 1.31 | 0.25 |
| Parietal lobe No. (%) | 151 (15.2) | 24 (16.9) | 0.16 | 0.69 |
| Occipital lobe No. (%) | 98 (9.9) | 12 (8.5) | 0.15 | 0.70 |
| Temporal lobe No. (%) | 401 (40.4) | 51 (35.9) | 0.87 | 0.35 |
| Insular lobe No. (%) | 81 (8.2) | 9 (6.3) | 0.35 | 0.56 |
| **Molecular and Pathological features** | | | | |
| WHO Grade 1/2/3/4 | 1/142/182/667 | 2/55/27/58 | 64.23 | 7.34×10^- 14***^ |
| WHO Grade 1 No. (%) | 1 (0.1) | 2 (1.4) | -- | -- |
| WHO Grade 2 No. (%) | 142 (14.3) | 55 (38.7) | -- | -- |
| WHO Grade 3 No. (%) | 182 (18.3) | 27 (19.0) | -- | -- |
| WHO Grade 4 No. (%) | 667 (67.2) | 58 (40.8) | -- | -- |
| GFAP+^a^ No. (%) | 978 (99.5) | 139 (100.0) | 0.00 | 1.00 |
| OLIG2+^a^ No. (%) | 887 (91.5) | 121 (89.0) | 0.69 | 0.41 |
| P53+^a^ No. (%) | 757 (78.0) | 100 (72.5) | 1.78 | 0.18 |
| IDH-1/ IDH-2+^a^ No. (%) | 156 (21.0) | 64 (51.6) | 50.86 | 9.94×10^- 13***^ |
| MGMT+^a^ No. (%) | 436 (51.7) | 72 (61.0) | 3.27 | 0.07 |
| EGFR+^a^ No. (%) | 67 (80.7) | 15 (60.0) | 3.45 | 0.06 |
| Chemoradiotherapy No. (%) | 865 (87.2) | 122 (85.9) | 0.09 | 0.77 |
| Ki-67^a^ > 20% No. (%) | 253 (26.4) | 29 (21.2) | 1.43 | 0.23 |
| **Follow-up data 1 month after initial surgery** | | | | |
| Postoperative KPS | 86.3 (15.8) | 88.2 (14.9) | 1.31 | 0.19 |
| Postoperative clinical neurological function examination^a^ | 313/504/124/40/7 | 51/67/16/2/1 | 3.48 | 0.48 |
| obstacle 0 No. (%) | 313 (31.7) | 51 (37.2) | -- | -- |
| obstacle 1 No. (%) | 504 (51.0) | 67 (48.9) | -- | -- |
| obstacle 2 No. (%) | 124 (12.6) | 16 (11.7) | -- | -- |
| obstacle 3 No. (%) | 40 (4.0) | 2 (1.5) | -- | -- |
| obstacle 4 No. (%) | 7 (0.7) | 1 (0.7) | -- | -- |
| ECOG Grade^a^ | 262/537/145/35/7 | 37/77/18/5/0 | 1.25 | 0.87 |
| grade 0 No. (%) | 262 (26.6) | 37 (27.0) | -- | -- |
| grade 1 No. (%) | 537 (54.5) | 77 (56.2) | -- | -- |
| grade 2 No. (%) | 145 (14.7) | 18 (13.1) | -- | -- |
| grade 3 No. (%) | 35 (3.5) | 5 (3.6) | -- | -- |
| grade 4 No. (%) | 7 (0.7) | 0 (0.0) | -- | -- |
| a: The majority of intracranial space - occupying lesion or intracranial tumor were discovered incidentally through accidental injury seeking medical attention or physical examination. Tumors of deep brain structures including Corpus Callosum, Thalamus, Basal ganglia, Cerebellum, Ventricle, Saddle area, Brain stem and other areas; GFAP+, glial fibrillary acidic protein positive; OLIG2+, oligodendrocyte transcription factor 2 overexpression; TP53+, tumor suppressor gene p53 mutation; IDH - 1/ IDH - 2+, isocitrate dehydrogenase 1 or isocitrate dehydrogenase 2 mutation; Ki - 67, cell proliferation antigen Ki - 67; TERT+, telomerase reverse transcriptase promoter mutation; 1p/19q codeletion, complete deletion of both the short arm of chromosome 1 (1p) and the long arm of chromosome 19 (19q); MGMT+, O - 6 - methylguanine - DNA methyltransferase promoter methylation; EGFR+, epidermal growth factor receptor mutation. Postoperative clinical neurological function examination is an assessment of the patient's neurological function by clinicians based on family and work autonomy and neurological dysfunction examination, and is divided into five grades from no to severe disorders according to severity. ECOG Grade refers to a scoring system that measures the extent to which cancer affects a patient's daily living abilities (performance status) on a scale ranging from 0 (fully active) to 5 (dead). *: p < 0.05, **: p < 1×10^-2^,***: p < 1×10^-3^. | | | | |

| **Table S2 Percentage of Missing data on variables with single and repeat resection patients** | | | |
| --- | --- | --- | --- |
| **Demographic and Clinical Data** | **Single resection**  **(n = 140)** | **Reoperation**  **(n = 140)** | **Total** |
| Age at Diagnosis (%) | 0 (0) | 0 (0) | 0 (0) |
| Sex male No. (%) | 0 (0) | 0 (0) | 0 (0) |
| Preoperative KPS | 0 (0) | 0 (0) | 0 (0) |
| **Chief complaints** | | | |
| Headache No. (%) | 0 (0) | 0 (0) | 0 (0) |
| Dizziness No. (%) | 0 (0) | 0 (0) | 0 (0) |
| Nausea No. (%) | 0 (0) | 0 (0) | 0 (0) |
| Vomiting No. (%) | 0 (0) | 0 (0) | 0 (0) |
| Seizure No. (%) | 0 (0) | 0 (0) | 0 (0) |
| Intracranial Space - Occupying Lesion or Intracranial Tumor No. (%) | 0 (0) | 0 (0) | 0 (0) |
| Impaired Consciousness No. (%) | 0 (0) | 0 (0) | 0 (0) |
| Muscle Weakness No. (%) | 0 (0) | 0 (0) | 0 (0) |
| Limb Numbness No. (%) | 0 (0) | 0 (0) | 0 (0) |
| Speech Disorder No. (%) | 0 (0) | 0 (0) | 0 (0) |
| Memory Deterioration No. (%) | 0 (0) | 0 (0) | 0 (0) |
| Visual Impairment No. (%) | 0 (0) | 0 (0) | 0 (0) |
| Lethargy No. (%) | 0 (0) | 0 (0) | 0 (0) |
| History of Head Trauma No. (%) | 0 (0) | 0 (0) | 0 (0) |
| **Tumor Characteristics and Surgical Features** | | | |
| Frontal lobe No. (%) | 0 (0) | 0 (0) | 0 (0) |
| Parietal lobe No. (%) | 0 (0) | 0 (0) | 0 (0) |
| Occipital lobe No. (%) | 0 (0) | 0 (0) | 0 (0) |
| Temporal lobe No. (%) | 0 (0) | 0 (0) | 0 (0) |
| Insular lobe No. (%) | 0 (0) | 0 (0) | 0 (0) |
| Tumors of deep cerebral structures No. (%) | 0 (0) | 0 (0) | 0 (0) |
| Extent of resection No. (%) | 36 (25.7) | 43 (30.7) | 79 (28.2) |
| **Molecular and Pathological features** | | | |
| WHO Grade 1/2/3/4 No. (%) | 0 (0) | 0 (0) | 0 (0) |
| GFAP+ No. (%) | 1 (0.7) | 3 (2.1) | 4 (1.4) |
| OLIG2+ No. (%) | 3 (2.1) | 1 (0.7) | 4 (1.4) |
| TP53+ No. (%) | 1 (0.7) | 1 (0.7) | 2 (0.7) |
| IDH - 1/ IDH - 2+ No. (%) | 9 (6.4) | 0 (0) | 9 (3.2) |
| MGMT+ No. (%) | 7 (5) | 0 (0) | 7 (2.5) |
| Ki - 67 > 20% No. (%) | 1 (0.7) | 1 (0.7) | 2 (0.7) |
| **Follow-up data 1 month after initial surgery** | | | |
| Postoperative KPS No. (%) | 0 (0) | 5 (3.6) | 5 (1.8) |
| Chemoradiotherapy No. (%) | 0 (0) | 0 (0) | 0 (0) |
| Postoperative clinical neurological function examination No. (%) | 0 (0) | 5 (3.6) | 5 (1.8) |
| ECOG Grade No. (%) | 0 (0) | 5 (3.6) | 5 (1.8) |
| Progression-free survivalb (PFS) No. (%) | 0 (0) | 0 (0) | 0 (0) |
| *: The tables are all presented as missing proportions, and the numbers in parentheses are missing proportions within groups. | | | |

| **Table S3 KPS scores before and after reoperation: LOCF sensitivity analysis and available-case analyses** | | | |
| --- | --- | --- | --- |
| **Performance status comparison (KPS)** | **Reoperation**  **(n = 140)** | **t** | **p value** |
| **KPS before reoperation** |  |  |  |
| Mean (SD) | 78.2 (10.3)^a^ | 8.98 | 6.04×10^-13***b^ |
| **KPS 1 month after reoperation** |  |  |  |
| Mean (SD) | 84.0 (18.4)^a^ | 2.27 | 0.03^*c^ |
|  | 85.3 (18.5)^d^ |  |  |
| **KPS 6 month after reoperation** |  |  |  |
| Mean (SD) | 80.2 (20.7)^a^ | 0.75 | 0.45^e^ |
|  | 82.5 (20.8)^f^ |  |  |
| ^a^Calculated from 65 patients with complete KPS data before reoperation and at 1 and 6 months after reoperation (after LOCF imputation of missing pre-reoperation KPS values).  ^b^from KPS scores before reoperation compared to KPS before initial surgery in 65 patients.  ^c^from KPS scores before reoperation compared to short-term after reoperation in 65 patients.  ^d^Calculated from 133 patients with complete KPS data at 1 month after reoperation.  ^e^from KPS scores before reoperation compared to long-term after reoperation in 65 patients.  ^f^Calculated from 117 patients with complete KPS data at 6 months after reoperation.  *: p < 0.05, **: p < 1×10^-2^, ***: p < 1×10^-3^ | | | |

| **Table S4 Univariable and Multivariable Cox Analysis of the Reoperation Population (n=133)** | | | | | | |
| --- | --- | --- | --- | --- | --- | --- |
| **Variables** | **Univariable Analysis** | | | **Multivariable Analysis** | | |
|  | **HR** | **95% CI** | **p value** | **HR** | **95% CI** | **p value** |
| Age at Diagnosis (Years) | 1.02 | 0.99 - 1.03 | 0.07 | 1.00 | 0.98 - 1.02 | 0.93 |
| Sex male/female | 1.68 | 1.10 - 2.56 | 0.02^*^ | 1.93 | 1.20 - 3.10 | 7.00×10^- 3**^ |
| WHO Grade 4 yes/no | 4.76 | 2.86 - 7.92 | 1.79×10 ^- 9***^ | 1.23 | 0.75 - 2.02 | 0.41 |
| Olig2+ yes/no | 0.22 | 0.12 - 0.42 | 1.26×10 ^- 6***^ |  |  |  |
| TP53+ yes/no | 1.37 | 0.86 - 2.18 | 0.19 | 1.14 | 0.59 - 2.21 | 0.69 |
| IDH - 1/ IDH - 2+ yes/no | 0.54 | 0.35 - 0.84 | 6.77×10 ^- 3**^ | 1.16 | 0.70 - 1.93 | 0.56 |
| MGMT+ yes/no | 1.34 | 0.83 - 2.13 | 0.22 | 1.34 | 0.84 - 2.12 | 0.22 |
| EGFR+ yes/no | 1.33 | 0.52 - 3.39 | 0.56 |  |  |  |
| Chemoradiotherapy yes/no | 0.67 | 0.36 - 1.23 | 0.20 |  |  |  |
| Ki - 67 > 20% yes/no | 2.35 | 1.45 - 3.81 | 5.37×10 ^- 4***^ | 1.17 | 0.60 - 2.30 | 0.64 |
| Interval between operations (days) |  |  |  |  |  |  |
| days 365-730 vs days <365 |  |  |  | 0.55 | 0.30 - 0.99 | 0.05^*^ |
| days > 730 vs days <365 |  |  |  | 0.10 | 0.05 - 0.18 | <0.001^***^ |
| Postoperative Disturbed sleep | 4.32 | 1.98 - 9.44 | 2.44×10 ^- 4***^ | 0.30 | 0.08 -1.18 | 0.09 |
| Postoperative general ability | 2.84 | 1.33 - 6.09 | 7.16×10 ^- 3**^ | 1.09 | 0.92 - 1.28 | 0.32 |
| The time interval between two operations was divided into less than 365 days, 365-730 days and more than 730 days, and the univariate analysis of this variable was not done because the PH hypothesis was not satisfied.*: p < 0.05, **: p < 1×10-2, ***: p < 1×10-3 | | | | | | |

| **Table S5 Multivariable Logistic Regression Models for Predicting Survival Benefit After Reoperation at Different Thresholds** | | | |
| --- | --- | --- | --- |
| **Variables** | **OR** | **95% CI** | **p value** |
| **Survival benefit greater than 3 months** | | | |
| Age at Diagnosis (Years) | 1.04 | (0.98, 1.09) | 0.20 |
| Sex male/female | 0.61 | (0.18, 2.03) | 0.42 |
| Interval between operations (days) | 1.002 | (1.001, 1.003) | <0.001^***^ |
| MGMT+ yes/no | 2.55 | (0.82, 7.93) | 0.11 |
| TP53+ yes/no | 0.66 | (0.18, 2.38) | 0.52 |
| IDH-1/ IDH-2+ yes/no | 0.26 | (0.06, 1.07) | 0.06 |
| Ki-67 > 20% yes/no | 0.90 | (0.19, 4.24) | 0.90 |
| WHO Grade 3 vs Grade 2 | 0.80 | (0.16, 3.91) | 0.78 |
| WHO Grade 4 vs Grade 2 | 0.83 | (0.15, 4.53) | 0.83 |
| **Survival benefit greater than 6 months** | | | |
| Age at Diagnosis (Years) | 1.05 | (0.99, 1.12) | 0.12 |
| Sex male/female | 0.37 | (0.09, 1.59) | 0.18 |
| Interval between operations (days) | 1.004 | (1.002, 1.006) | <0.001^***^ |
| MGMT+ yes/no | 2.56 | (0.67, 10.00) | 0.17 |
| TP53+ yes/no | 0.31 | (0.06, 1.56) | 0.16 |
| IDH-1/ IDH-2+ yes/no | 0.14 | (0.02, 1.04) | 0.05 |
| Ki-67 > 20% yes/no | 1.85 | (0.25, 14.29) | 0.55 |
| Lethargy^a^ | 0.02 | (0.0004, 1.25) | 0.06 |
| WHO Grade 3 vs Grade 2 | 0.43 | (0.06, 2.94) | 0.39 |
| WHO Grade 4 vs Grade 2 | 0.50 | (0.04, 5.88) | 0.59 |
| Limb numbness^a^ | 2.08 | (0.87, 5.00) | 0.10 |
| **Survival benefit greater than 9 months** | | | |
| Age at Diagnosis (Years) | 1.02 | (0.97, 1.07) | 0.45 |
| Sex male/female | 0.50 | (0.15, 1.65) | 0.25 |
| Interval between operations (days) | 1.003 | (1.001, 1.004) | <0.001^***^ |
| MGMT+ yes/no | 1.02 | (0.33, 3.17) | 0.97 |
| TP53+ yes/no | 0.92 | (0.26, 3.20) | 0.89 |
| IDH-1/ IDH-2+ yes/no | 0.23 | (0.05, 1.07) | 0.06 |
| Ki-67 > 20% yes/no | 0.36 | (0.07, 1.90) | 0.23 |
| WHO Grade 3 vs Grade 2 | 2.05 | (0.43, 9.72) | 0.37 |
| WHO Grade 4 vs Grade 2 | 1.40 | (0.23, 8.29) | 0.71 |
| **Survival benefit greater than 12 months** | | | |
| Age at Diagnosis (Years) | 0.99 | (0.94, 1.04) | 0.57 |
| Sex male/female | 0.44 | (0.13, 1.43) | 0.17 |
| Interval between operations (days) | 1.003 | (1.002, 1.004) | <0.001^***^ |
| MGMT+ yes/no | 0.70 | (0.22, 2.19) | 0.54 |
| TP53+ yes/no | 1.08 | (0.31, 3.81) | 0.90 |
| IDH-1/ IDH-2+ yes/no | 0.30 | (0.07, 1.34) | 0.11 |
| Ki-67 > 20% yes/no | 0.42 | (0.08, 2.27) | 0.31 |
| WHO Grade 3 vs Grade 2 | 1.82 | (0.39, 8.45) | 0.44 |
| WHO Grade 4 vs Grade 2 | 1.87 | (0.31, 11.26) | 0.49 |
| a: Lethargy and Limb numbness are preoperative patient self-reported symptoms *: p < 0.05, **: p < 1×10^-2^, ***: p < 1×10^-3^. | | | |

| **Table S6 Multivariable Logistic Regression Models for Predicting Survival Benefit After Reoperation at Different Thresholds in glioblastoma subset** | | | |
| --- | --- | --- | --- |
| **Variables** | **OR** | **95% CI** | **p value** |
| **Survival benefit greater than 3 months** | | | |
| Age at Diagnosis (Years) | 0.98 | (0.87, 1.11) | 0.74 |
| Sex male/female | 18.49 | (0.35, 982.82) | 0.15 |
| Interval between operations (days) | 1.007 | (1.000, 1.013) | 0.05^*^ |
| MGMT+ yes/no | 24.57 | (1.40, 431.66) | 0.03^*^ |
| **Survival benefit greater than 6 months** | | | |
| Age at Diagnosis (Years) | 0.96 | (0.87, 1.05) | 0.38 |
| Sex male/female | 7.69 | (0.46, 100.00) | 0.16 |
| Interval between operations (days) | 1.008 | (1.001, 1.012) | 0.01^*^ |
| TP53+ yes/no | 0.39 | (0.04, 3.70) | 0.41 |
| Limb numbness^a^ | 1.41 | (0.0006, 12.50) | 0.32 |
| **Survival benefit greater than 9 months** | | | |
| Age at Diagnosis (Years) | 1.00 | (0.94, 1.06) | 0.98 |
| Sex male/female | 1.57 | (0.32, 7.73) | 0.58 |
| Interval between operations (days) | 1.003 | (1.001, 1.006) | <0.01** |
| **Survival benefit greater than 12 months** | | | |
| Age at Diagnosis (Years) | 0.96 | (0.91, 1.03) | 0.26 |
| Sex male/female | 1.52 | (0.29, 7.82) | 0.62 |
| Interval between operations (days) | 1.003 | (1.001, 1.005) | <0.01** |
| a: Limb numbness is preoperative patient self-reported symptoms *: p < 0.05, **: p < 1×10^-2^, ***: p < 1×10^-3^. | | | |

**Figure S1**

**Figure S1 Flow chart of the study**

**Figure S2**

**Figure S2 Kaplan-Meier survival curve between the reoperation and non-reoperation groups in the subspecialty and non-subspecialty subgroups**

Parts (a) and (b) show the survival curves for reoperations performed by a subgroup of subspecialists (a) and non-subspecialists (b) at the hospital, respectively, where the physician's belonging to a subspecialty has strict categorization criteria at that hospital.

**Figure S3**

**Figure S3 Forest plots of hazard ratios and 95% CIs for reoperation survival rate by different surgeons involved in the cohort**

Part (a) shows the HRs of reoperations performed by the 10 primary surgeons in the matched cohort, while parts (b) shows the HRs of reoperations performed by different surgeons in the glioblastoma subgroup.

**Figure S4**

**Figure S4 Kaplan-Meier survival curve between the reoperation and non-reoperation groups in the same primary surgeon subgroup**

Tumor resections in 30 glioma patients in the subgroup were performed by one of our leading neurosurgeons.

**Figure S5**

**Figure S5 Kaplan-Meier survival curve for WHO grades in multivariate COX analysis**

The WHO classification uses pathological information after the first surgery.

**Figure S6**

**Figure S6 A novel scoring system to predict survival benefit beyond 6 months for glioma patients**

A scoring system based on logistic prediction model was developed, incorporating demographic, molecular and chief complaint information as clinical variables available prior to reoperation. In this illustrative case, a female patient, with an interval of 1500 days between surgeries, classified as WHO Grade 2, and experiencing no postoperative lethargy, computes her comprehensive score using the outlined Points: 43 + 7.6 + 7.75 + 30 = 88.35. The derived total score correlates with the probability of survival benefit beyond 6 months, as indicated at the bottom. Specifically, the probability of survival benefit extending beyond 6 months after reoperation surpasses 70% for this patient.

**Figure S7**

**Figure S7 A novel scoring system to predict survival benefit beyond 6 months for glioblastoma patients**

A scoring system based on logistic prediction model was developed, incorporating demographic, molecular and chief complaint information as clinical variables available prior to reoperation.
